# History of the Florence Nightingale Ward in the King’s College Hospital

**Published:** 1868-07

**Authors:** 


### History of the Florence Nightingale Ward in the King's College Hospital.

The author commenced by relating the circumstances under which the Nightingale Ward was founded, for the purpose of instructing duly-qualified midwives to be employed in attending on the poor under proper medical supervision. He referred to the condition upon which Dr. Arthur Farre, at that time physician accoucheur to the hospital, had insisted, before consenting to take charge of the ward, and described the arrangements for securing, as far as possible, the safety of the patients. The most elaborate precautions had been taken by Dr. Farre for the purpose. The long ward was only employed for convalescent patients. There were two separate delivery wards, which were used alternately for three weeks at a time, and in the interval the empty room was thoroughly cleansed and disinfected. Each patient had 3200 feet of breathing air. All students engaged in dissecting, or in attending the surgical practice of the hospital, were prohibited from entering the ward. Mr. Rowling then proceeded to give the statistics of the deliveries, and showed that, in spite of every care, the mortality had increased each year; the average mortality since the ward was opened having been 1 in 28 9 cases. He described the causes of two deaths that had occurred and their most prominent symptoms, and finally mentioned that this great mortality had determined the hospital authorities to close the department altogether.

Dr. Barnes said that no paper was more deserving of record in the Transactions than this, in order that it might stand as a warning against the repetition of the most disastrous experiment related. It had been said that history repeats itself; but why did history repeat itself? Simply because her plainest lessons were willfully disregarded. Was it necessary, at the sacrifice of the lives of so many women, to repeat an experiment which ample experience in every country had over and over again proved to be so fatal? He felt bound to say that this tendency to repeat a fatal mistake was more the fault of the lay members of society than of medical men. He did not suppose that any physician in the room would now advocate the establishment of a lying-in ward in a general hospital. Dr. Farre had never approved of it. He himself had strenuously resisted a proposition at one time contemplated to establish a similar ward in the new St. Thomas's. A lying-in hospital was bad enough, but a lying-in ward attached to a general hospital was a sin against humanity. Was it not shocking to offer in the name of charity to poor women looking to you for help, a succor that would only too probably be charged with death. So deeply had the mortality of lying-in hospitals, even of those constructed with every care that modern research could devise, impressed many of the most eminent men in Paris, that, the expediency of suppressing these hospitals and of substituting home-midwifery was now admitted. In his conversations with continental professors he had found few who contended that hospitals were desirable for the patients. The general argument for hospitals was that they were necessary for the purposes of instruction. Well, we taught midwifery here and we saved our women.

Dr. Graily Hewitt wished to make one remark on this subject. Lying-in hospitals, as they had been organized up to the present time, were most undoubtedly objectionable. The secret of the successful treatment of lying-in cases was isolation. The moment cases were congregated together in one apartment, puerperal fever was likely to be generated. If the patients were isolated from each other by suitable means, and in suitable buildings, there was no reason why the mortality should be higher in a lying-in hospital than elsewhere, but in the existing hospitals these precautions had not been attended to.—*Lancet.*

